# High-performance binder-free supercapacitor electrode by direct growth of cobalt-manganese composite oxide nansostructures on nickel foam

**DOI:** 10.1186/1556-276X-9-492

**Published:** 2014-09-13

**Authors:** Shulan Jiang, Tielin Shi, Hu Long, Yongming Sun, Wei Zhou, Zirong Tang

**Affiliations:** 1 State Key Laboratory of Digital Manufacturing Equipment and Technology, Huazhong University of Science and Technology, 1037 Luoyu Road, Wuhan 430074, China; 2 Wuhan Jiawei Photovoltaic Lighting Co. LTD, Wuhan, China

**Keywords:** Cobalt-manganese composite oxide, Hierarchical nanosheets, Binder-free, Supercapacitor electrode

## Abstract

A facile approach composed of hydrothermal process and annealing treatment is proposed to directly grow cobalt-manganese composite oxide ((Co,Mn)_3_O_4_) nanostructures on three-dimensional (3D) conductive nickel (Ni) foam for a supercapacitor electrode. The as-fabricated porous electrode exhibits excellent rate capability and high specific capacitance of 840.2 F g^-1^ at the current density of 10 A g^-1^, and the electrode also shows excellent cycling performance, which retains 102% of its initial discharge capacitance after 7,000 cycles. The fabricated binder-free hierarchical composite electrode with superior electrochemical performance is a promising candidate for high-performance supercapacitors.

## Background

Due to the depletion of fossil fuels and increasingly serious environmental pollution, there has been an urgent demand for advanced and high-performance energy storage devices to satisfy the needs of modern society and emerging ecological concerns [1,2]. Supercapacitors, also called electrochemical capacitors, have attracted a great deal of attention due to their excellent performance like high power density, long cycle life, high reliability, etc. [3-5]. They store energy through ion adsorption at the electrode/electrolyte interface or based on faradaic redox reactions by using high-energy electrode materials such as metal oxides, metal-doped carbons, or conductive polymers [6]. Growing interest has concentrated on the metal-oxide nanostructures with excellent electrochemical performance in recent years. Simple binary metal oxide materials such as manganese dioxide (MnO_2_) [7-9], cobaltosic oxide (Co_3_O_4_) [10,11], nickel oxide (NiO) [12,13], and ruthenium oxide (RuO_2_) [14,15] have been widely studied as supercapacitor electrodes and show good electrochemical performance. Meanwhile, ternary metal oxides with two different metal cations like NiCo_2_O_4_[16-18], ZnCo_2_O_4_[19,20], and MnCo_2_O_4_[21,22] have also garnered attentions in recent years due to their promising applications in energy storage fields [22]. The coupling of two metal species could make oxidation-state-rich redox reactions which are essential for pseudocapacitor and various combinations of the cations during the charging-discharging process, and the tunable stoichiometric/non-stoichiometric compositions of the mixed transition metal oxides could provide great opportunities to manipulate the physical/chemical properties [17,22-24].

Ternary Co-Mn oxides have been widely studied as lithium-ion battery electrodes in recent reports [22,23,25,26], which demonstrates excellent electrochemical performance. For example, Li et al. [22] prepared MnCo_2_O_4_ quasi-hollow microspheres which maintained remarkable reversible capacities of 755 mA h g^-1^ at a current density of 200 mA g^-1^ after 25 cycles when used as an anode material for lithium ion batteries. Recently, efforts have been devoted to the study of Co-Mn oxide structures as supercapacitor electrode materials [21,24,27-29]. Pure MnCo_2_O_4_[21,24] and MnCo_2_O_4.5_[27] nanostructures have been synthesized through the hydrothermal method or solvothermal technique and tested as supercapacitor electrodes, showing potential applications in supercapacitors. Co-Mn composite oxide structures have also been fabricated through the thermally decomposing method [28] or electroless electrolytic technique [29], showing improved electrochemical performance compared with the pure MnCo_2_O_4_ and MnCo_2_O_4.5_ nanostructures.

In this work, a facile approach was proposed to fabricate binder-free Co-Mn composite electrodes ((Co,Mn)_3_O_4_ nanostructures/Ni foam) for supercapacitors. The method mainly consisted of two steps. Firstly, Co-Mn composite oxide hierarchical nanosheets were directly grown on three-dimensional (3D) Ni foam substrates through a hydrothermal process. Then a post-annealing treatment was conducted at 400°C in air. Directly growing nanostructured arrays on conductive substrates without the use of a binder or an additive can avoid the ‘dead surface’ in conventional slurry-derived electrodes and allow for more efficient charge and mass exchange. The Co-Mn composite oxide ((Co,Mn)_3_O_4_) nanostructures/Ni foam electrode was evaluated as a supercapacitor electrode and showed superior electrochemical performance.

## Methods

All the reagents used in the experiment were of analytical grade. The facile approach was proposed to grow Co-Mn composite nanostructures on Ni foam through a hydrothermal method followed by annealing treatment. In a typical process, commercially available Ni foam was used as current collector and treated with acetone, hydrochloric acid, deionized water, and ethanol in sequence. To obtain a homogeneous precursor solution, 0.338 g manganese sulfate (MnSO_4_.H_2_O), 0.291 g cobaltous nitrate (Co(NO_3_)_2_.6H_2_O), 0.721 g urea, and 0.444 g ammonium fluoride (NH_4_F) were dissolved into the mixed solvent of 40 ml deionized water and 40 ml ethanol under magnetic stirring. The role of NH_4_F in the formation of different morphologies has been investigated by changing the weight of NH_4_F such as 0.222 and 0.888 g. After drying, the well-cleaned Ni foam was immersed in the precursor solution. Then the solution was transferred into a Teflon-lined stainless steel autoclave and heated at 120°C for 8 h. After reaction and cooled to room temperature, the substrate was taken out and cleaned with ethanol and deionized water before being dried in air. The dried sample was then annealed in air at 400°C with the heating rate of 1°C min^-1^ and kept for 4 h to obtain the Co-Mn composite oxide hierarchical structures. Both the Co-Mn composite oxide hierarchical structures/Ni foam and bare Ni foam were weighed in a high-precision analytical balance (Sartorius, Bradford, MA, USA), respectively. The loading density of the Co-Mn nanostructures in the sample is calculated as around 0.80 mg cm^-2^.

The morphologies were observed with scanning electron microscopy (SEM, SIRION200, Hillsboro, OR, USA) coupled with an energy-dispersive X-ray (EDX, Oxford Instrument, Abingdon, UK). Transmission electron microscopy (TEM) observation was carried out on a JEOL 2100 F microscope (JEOL Ltd., Tokyo, Japan). The nanostructures scratched down from the nickel foam were characterized by X-ray diffraction (XRD) analysis using Bruke D8-Advance (Bruker AXS, Inc., Madison, WI, USA). The specific surface area of the Co-Mn oxide nanostructures was determined by the Brunauer-Emmett-Teller (BET) equation and the pore size distribution was obtained through the BJH method. X-ray photoelectron spectroscopy (XPS) measurements were performed on a VG MultiLab 2000 system with a monochromatic Al Ka X-ray source (ThermoVG Scientific, East Grinstead, West Sussex, UK). The Co-Mn composite oxide nanostructures/Ni foam electrode was evaluated for a high-performance supercapacitor by the three-electrode system in 1 M KOH aqueous solution. The three-electrode assembly was constructed using the sample as the working electrode, a saturated calomel electrode (SCE) as the reference electrode, and a Pt foil as the counter electrode. The close contact of Co-Mn nanostructures on the current collector Ni foam allows for efficient charge transport, and waives the need for adding ancillary conducting material or binders. Cyclic voltammetry (CV) and electrochemical impedance spectroscopy (EIS) tests were conducted on an Autolab work station (PGSTAT-302 N, Eco Echemie B.V. Company, Utrecht, Netherlands). The electrochemical performance of pure Ni foam after being annealed at the same conditions as those for Co-Mn composite oxide nanostructures/Ni foam was tested by the CV technique. Galvanostatic charging/discharging and cycling tests were conducted using a battery measurement system (LAND CT2001A, Wuhan LAND Electronics, Wuhan, China).

## Results and discussion

**Figure 1:**
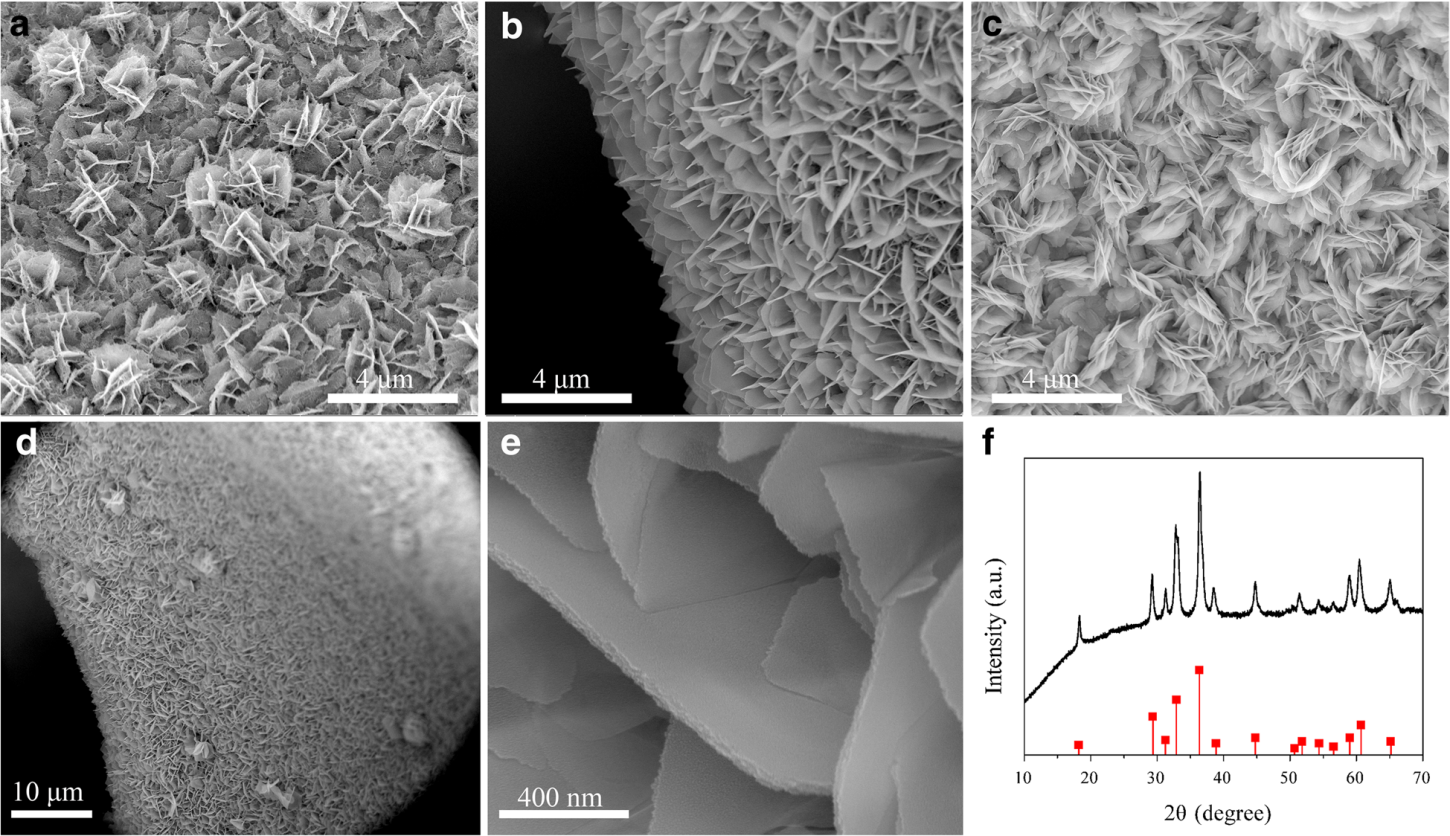
**SEM images and XRD patterns.** SEM images of the products obtained with different masses of NH_4_F: **(a)** 0.222 g, **(b)** 0.444 g, **(c)** 0.888 g. **(d,e)** SEM images of the Co-Mn composite oxide nanosheets obtained with 0.444 g NH_4_F. **(f)** XRD patterns of Co-Mn composite oxide nanosheets scratched down from the nickel foam.

The morphologies of the fabricated Co-Mn composite nanostructures were obtained through SEM. As shown in Figure 1a,b,c, different morphologies of hierarchical nanostructures had been obtained through adding different mass of NH_4_F. When the weight of NH_4_F was 0.222 g, sparse flower-like nanosheets could be observed shown in Figure 1a. When the weight was increased to 0.444 g, equally distributed nanosheets were formed (Figure 1b). As the weight of NH_4_F was further adjusted to 0.888 g, highly dense and agglomerate nanostructures were formed, as shown in Figure 1c. It is proposed that the introduction of NH_4_F could activate the substrate and lead to rough nanoscale surface [30]. The activated surface would promote the nucleation and growth of the nanostructures, and these nanostructures would form interconnected sheets and aggregate with an excessive amount of NH_4_F as shown in Figure 1c. We chose the samples obtained by the process with the addition of 0.444 g NH_4_F for further study in this work, and SEM images of the nanostructure with different magnifications are shown in Figure 1d,e, respectively. It is clear that these nanosheets are intercrossed and interconnected with each other, which form intricate transportation networks. The nanosheets with the thickness of around 30 nm exhibit porous nanostructures and non-smooth surface at the edge as shown in Figure 1e. The XRD patterns of the nanostructures (Figure 1d,e) scratched from nickel foam are shown in Figure 1f. All the reflection peaks of the nanostructures match well with the tetragonal spinel (Co,Mn)_3_O_4_ (JCPDS Card no. 18–0408). Small peaks centered at 2*θ* = 36.36° and 65.18° were found, which can be ascribed to the (311) and (440) planes of (Co,Mn)_3_O_4_, indicating that the (Co,Mn)_3_O_4_ phase was formed [31].

**Figure 2:**
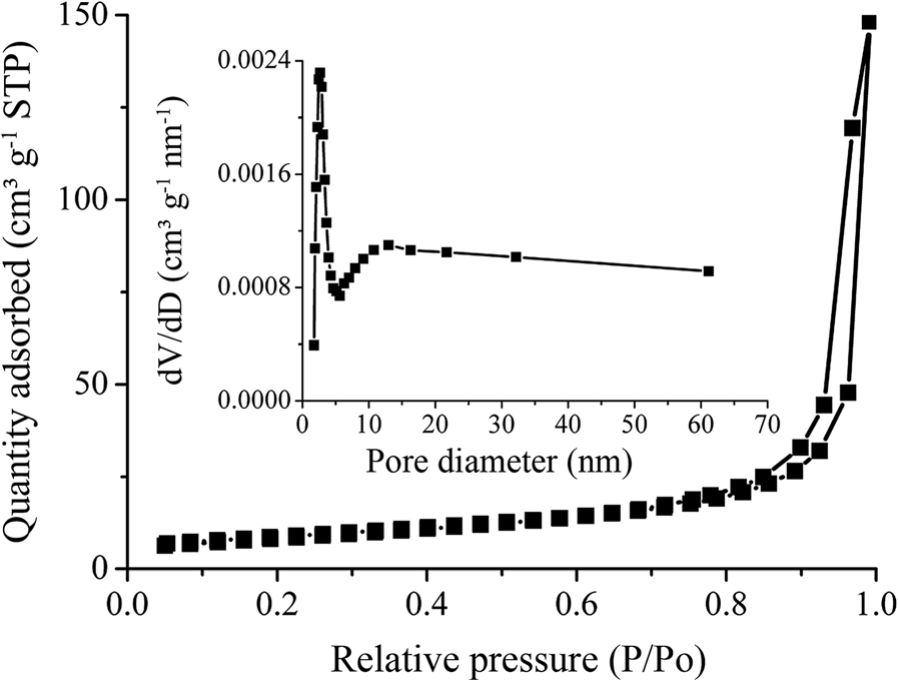
**N**_**2 **_**adsorption-desorption isotherms of Co-Mn oxide hierarchical architectures and corresponding pore size distribution curves.**

**Figure 3:**
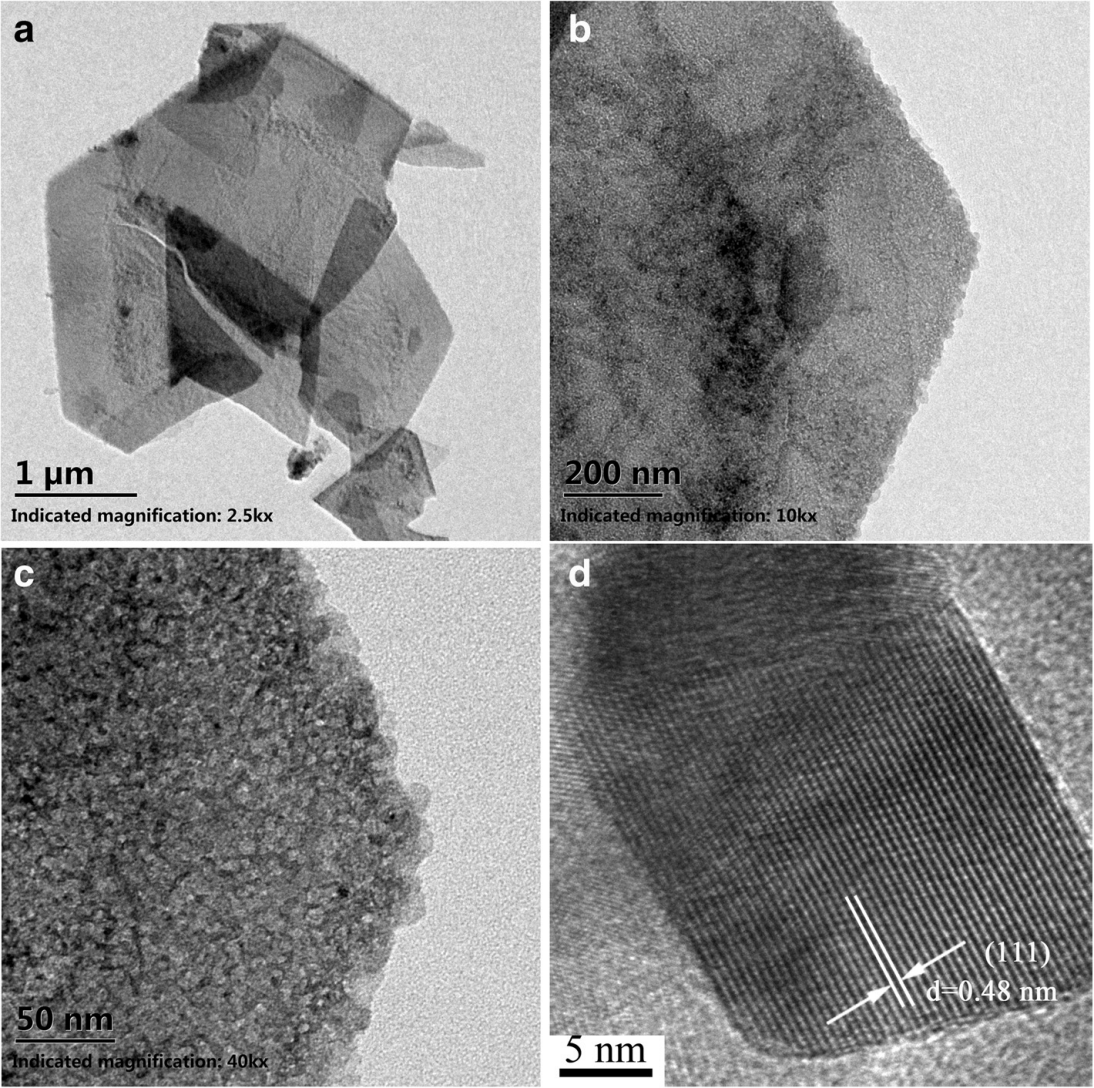
TEM images and HRTEM image of the Co-Mn oxide nanosheets scratched down from the nickel foam (a-d).

**Figure 4:**
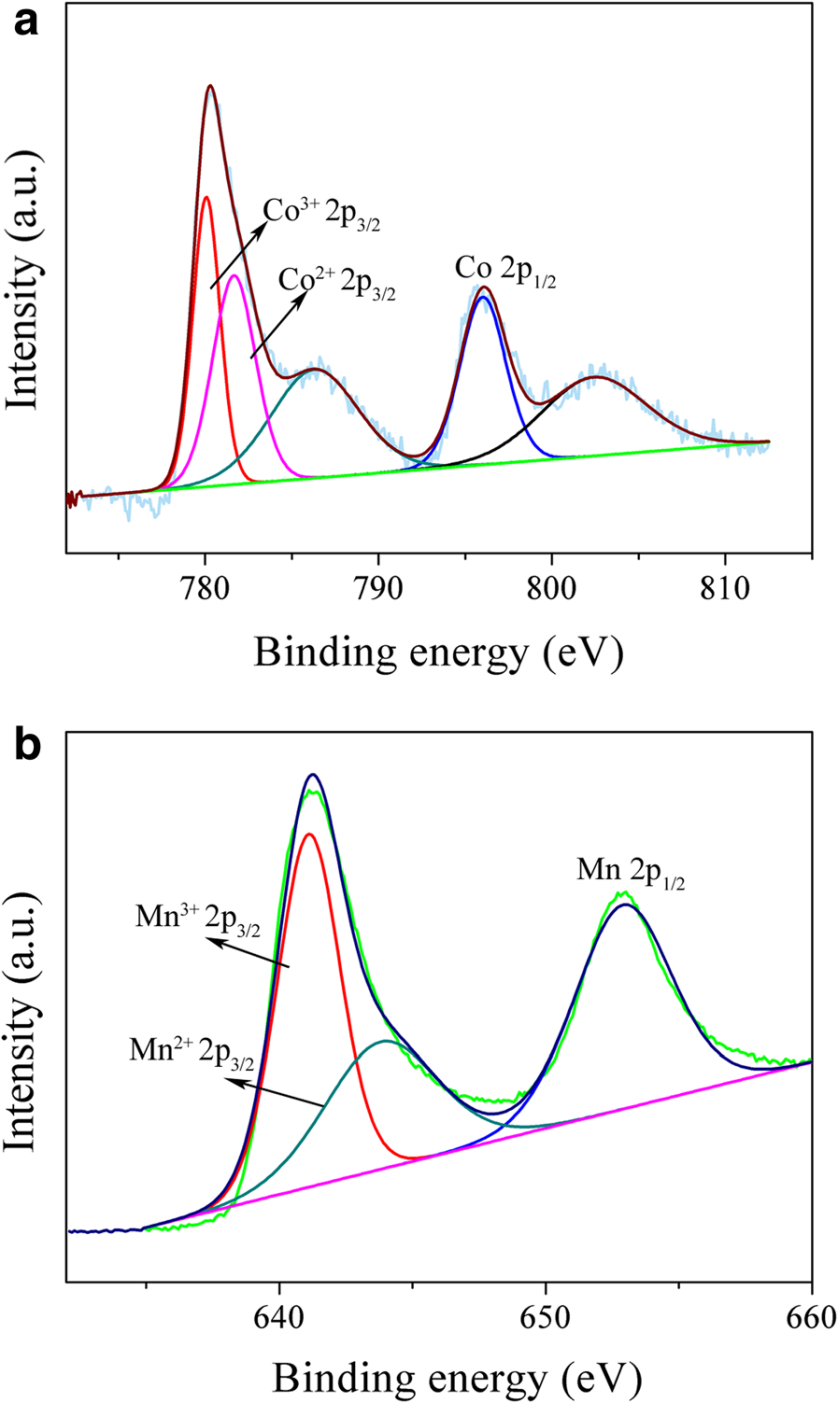
**XPS spectra of Co 2** ***p *** **(a) and Mn 2** ***p *** **for the Co-Mn composite oxide hierarchical nanosheets (b).**

The specific surface area of the structures was determined by N_2_ sorption measurement at 77 K. The hierarchical nanostructures show a BET surface area of 30.61 m^2^ g^-1^ as shown in Figure 2. The BET surface area of the Co-Mn oxide nanostructures is larger than those of the reported MnCo_2_O_4_ nanosheets (19 m^2^ g^-1^) [32] and porous MnCo_2_O_4.5_ hierarchical architectures (22.4 m^2^ g^-1^) [27]. This is probably due to the open pores formed by the nanoparticles in the Co-Mn oxide nanostructures, which resulted in a large surface area. The corresponding pore volume of the Co-Mn oxide hierarchical structures is about 0.23 cm^3^ g^-1^, while the pore volumes of the reported MnCo_2_O_4_ spinel oxide nanostructure synthesized by solvothermal technique are about 0.162 cm^3^ g^-1^ and 0.125 cm^3^ g^-1^ at 400°C and 300°C, respectively [24]. The porous Co-Mn composite oxide hierarchical nanosheets could offer a sufficient interface to facilitate the electrochemical reactions [33,34]. The TEM images of the Co-Mn composite nanosheets at different magnifications are shown in Figure 3a,b,c. It shows that the nanosheets were porous and composed of nanoparticles. The HRTEM image for the nanostructures (Figure 3d) indicated that the nanoparticles are highly crystallized. The lattice space of 0.48 nm corresponds to the (111) plane of the spinel-structured (Co, Mn)_3_O_4_, which is in good agreement with the calculated value from XRD. The surface electronic state and composition of the nanostructures were analyzed by XPS, as shown in Figure 4. The ratio of Mn and Co in the cobalt-manganese composite oxide nanostructures are about 2.48 according to the XPS result analysis. The Co 2*p* XPS spectra of the sample exhibit two main peaks at approximately 795.7 eV and approximately 780.2 eV, corresponding to the Co 2*p*_1/2_ and Co 2*p*_3/2_ spin-orbit peaks, respectively (Figure 4a). Two prominent shake-up satellite peaks (around 786.3 and 802.9 eV) are also observed, which show the presence of the Co^2+^[22]. The Mn 2*p* spectrum features two main spin-orbit lines of 2*p*_3/2_ at approximately 641.5 eV and 2*p*_1/2_ at approximately 653.2 eV (Figure 4b). The binding energies of the Mn^2+^ 2*p*_3/2_ and Mn^3+^ 2*p*_3/2_ are about 643.8 and 641.5 eV, respectively [31]. In conclusion, the solid-state redox couples Mn^3+^/Mn^2+^ and Co^3+^/Co^2+^ are present in these hierarchical structures, which is in agreement with the literature reported [21,22,31].

**Figure 5:**
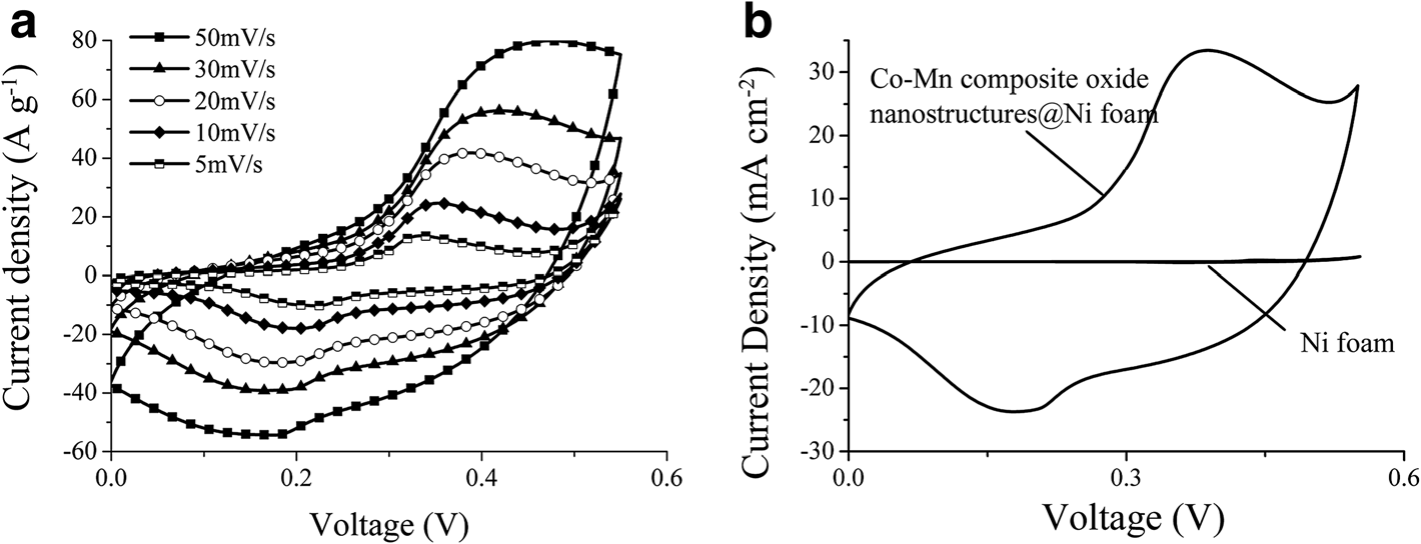
**CV curves at different rates and CV comparison between Ni foam and Co-Mn composite oxide nanostructures/Ni foam. (a)** CV curves at different scan rates recorded from Co-Mn composite oxide hierarchical structures/Ni foam electrode. **(b)** CV comparison of the treated Ni foam and Co-Mn composite oxide nanostructures/Ni foam at the scan rate of 20 mV s^-1^.

**Figure 6:**
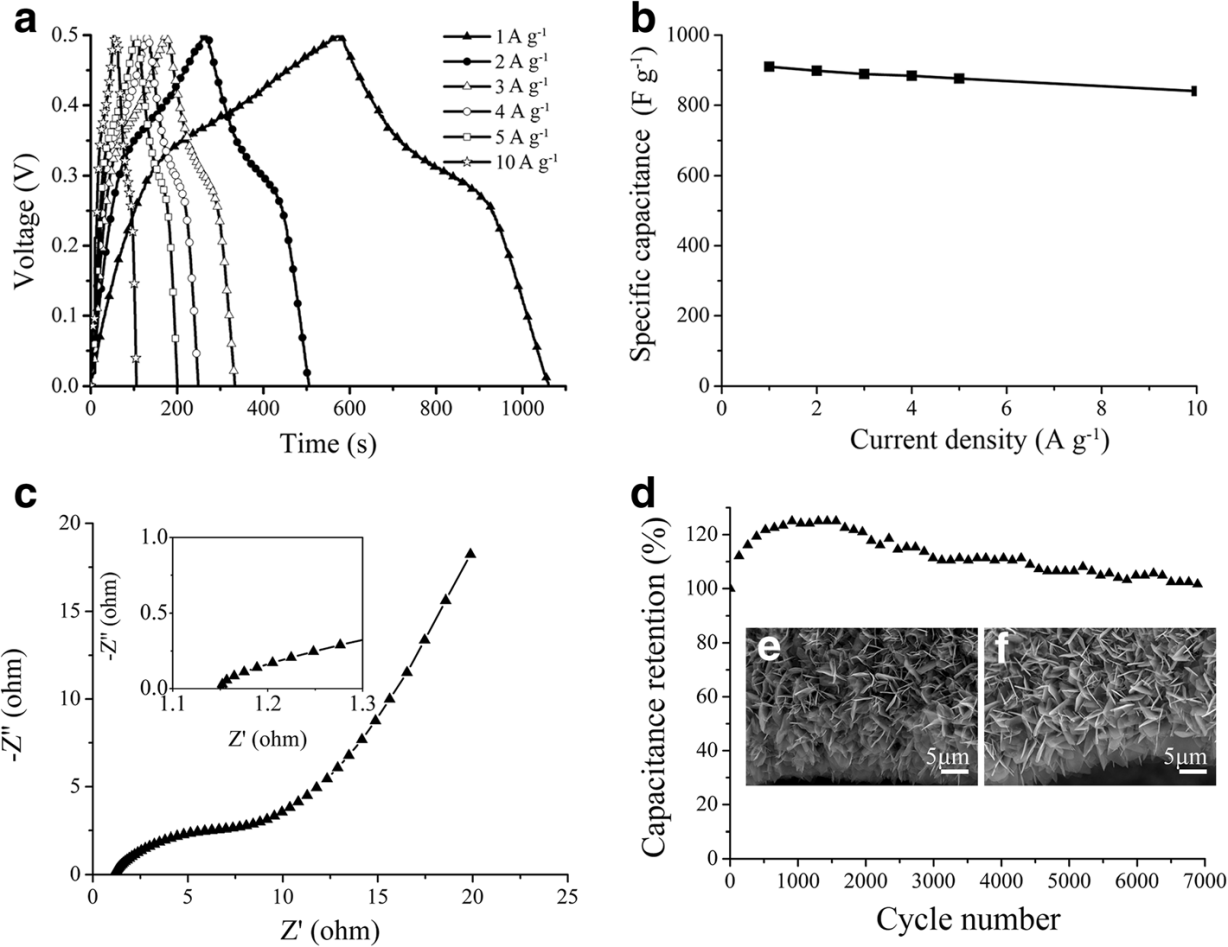
**Electrochemical properties of the Co-Mn composite oxide nanostructures/Ni foam electrode. (a)** Galvanostatic discharge-charge voltage profiles of the electrode at different current densities. **(b)** Specific capacitances at different current densities. **(c)** EIS test of the Co-Mn oxide nanosheets/Ni foam electrode. **(d)** Cycling performance of the Co-Mn oxide nanosheets/Ni foam electrode at a charge-discharge current density of 10 A g^-1^. **(e**,**f)** Morphologies of Co-Mn oxide hierarchical nanostructures before and after cycling tests, respectively.

The as-fabricated Co-Mn composite oxide ((Co,Mn)_3_O_4_) nanosheets/Ni foam was evaluated as a supercapacitor electrode. The electrochemical performance of the electrode was tested by the CV technique. The CV curves of the Co-Mn hierarchical nanosheets/Ni foam electrode in the potential region of 0 to 0.55 V (SCE) at different scan rates are shown in Figure 5a. The shape of CV curves with one oxidation peak and one reduction peak clearly reveals the pseudocapacitive characteristics [21]. Upon increasing the scan rate, the redox current increases; the anodic/cathodic peak shifts toward positive/negative potential, respectively, and the redox current increases. This phenomenon is caused by the kinetic irreversibility in the redox process due to polarization and ohmic resistance [35,36]. Figure 5b shows the CV comparison of the treated Ni foam and Co-Mn composite oxide nanostructures/Ni foam at the scan rate of 20 mV s^-1^, indicating that the nickel foam contributes little to total capacitance of the Co-Mn composite oxide nanostructures/Ni foam electrode. The electrochemical performance of the electrode was also evaluated by galvanostatic charge-discharge techniques. Figure 6a shows the charge-discharge curves of the electrode in a voltage range of 0 to 0.5 V at the current densities of 1 ~ 10 A g^-1^. The applied voltages of the electrode show good symmetry during charge-discharge for the total range of potential. The specific capacitance was calculated according to the following equation:

$$C=\frac{\mathrm{I\Delta t}}{\mathrm{m\Delta V}}$$

where *I* (A), Δ*t* (s), *m* (g), and Δ*V* (V) are the discharge current, discharge time, mass of the active materials, and the potential windows, respectively. The small capacitance of nickel foam could be neglected in the calculation of specific capacitance [28]. As shown in Figure 6b, the Co-Mn composite oxide structures/Ni foam electrode exhibits high specific capacitances of about 910.1, 898.6, and 889.2 F g^-1^ at relatively low current densities of 1, 2, and 3 A g^-1^, respectively. When the current density is increased to 10 A g^-1^, the specific capacitance of the electrode is around 840.2 F g^-1^, which keeps 92.3% of the specific capacitance at the current density of 1 A g^-1^. In comparison with literature reports, MnCo_2_O_4.5_ hierarchical architectures [27] with a specific capacitance of 151.2 F g^-1^ at the scan rate of 5 mV s^-1^, one-dimensional MnCo_2_O_4_ nanowire arrays [21] with a specific capacitance of 349.8 F g^-1^ at the current density of 1 A g^-1^, and Co-Mn oxide/carbon-nanofiber composite electrodes with a specific capacitance of 630 F g^-1^ at the scan rate of 5 mV s^-1^, this study demonstrates much higher specific capacitance of Co-Mn composites. The reported (Co,Mn)_3_O_4_ nanowires/Ni foam electrode exhibits a specific capacitance of 611 F g^-1^ at the current density of 2.38 A g^-1^ in 6.0 mol dm^-3^ KOH electrolyte [37]. The excellent rate capability and high specific capacitance of the as-fabricated Co-Mn composite oxide nanosheets/Ni foam mainly attribute to the unique 3D hierarchical porous structures of the Co-Mn composite oxide electrode [38]. The binder-free nanosheet structures can ensure good electric contact with the 3D Ni foam, and the open spaces between neighboring nanosheets allow for easy diffusion of the electrolyte, which is helpful for charging or discharging at a high current density. Moreover, the porous nature of the nanosheet structures will enhance the electrolyte/electrode contact area, shorten the ion diffusion distance, and accommodate the strain induced by the volume change during the redox reaction [38].

EIS measurement was also employed to characterize the composite electrodes over the frequency range from 0.1 to 10^6^ Hz as shown in Figure 6c. The intersection of the Nyquist plot on the real axis is related to the equivalent series resistance. The resistance of the hierarchical Co-Mn oxide nanosheets/Ni foam electrode is around 1.15 Ω, which is lower than that of the reported Co-Mn oxide hierarchical structure electrode (1.3 Ω) [27], indicating the improved charge transport properties of the electrode. The excellent rate capability and high specific capacitance of the electrode are also due to the relatively small ESR, which could improve the fast redox reaction [28]. The cycling performance of the electrode was tested at the current density of 10 A g^-1^ and the result is shown in Figure 6d. When the electrode was cycled up to 7,000 cycles, over 102% of its initial discharge capacitance is retained. In addition, during the first 1,000 cycles, the specific capacitance was increasing gradually, indicating an electroactivation process of the electrode under the given testing conditions (in a voltage range of 0 to 0.5 V, at the current density of 10 A g^-1^) [39]. Figure 6e,f shows the morphologies of the Co-Mn composite oxide hierarchical nanostructures before and after cycling tests, respectively. There are no obvious cracks and collapses on the hierarchical structures after being tested for 7,000 cycles, indicating good mechanical stability of the fabricated Co-Mn composite oxide hierarchical structures. The electrode with both long-term stability and superior electrochemical performance shows promising applications for high-performance supercapacitors.

## Conclusions

In summary, we have developed a facile approach to grow Co-Mn composite oxide ((Co,Mn)_3_O_4_) hierarchical nanosheets on 3D conductive Ni foam through a hydrothermal method together with a post-annealing treatment. The porous nanosheets/Ni foam electrode shows a high specific capacitance of 910.1 and 840.2 F g^-1^ at the current density of 1 and 10 A g^-1^, respectively. Over 102% of its initial discharge capacitance is retained after 7,000 cycling cycles at the current density of 10 A g^-1^. The outstanding electrochemical performance will undoubtedly make the Co-Mn composite oxide hierarchical nanostructures a promising candidate for high performance supercapacitor.

## Competing interests

The authors declare that they have no competing interests.

## Authors’ contributions

SJ and ZT designed the experiment. SJ and LH performed the experiments. TS, WZ, and YS contributed to material analysis and electrochemical performance analysis. SJ and ZT co-wrote the paper. All authors read and approved the final manuscript.
